# Multicenter validation of a galactomannan chemiluminescence immunoassay for the diagnosis of pulmonary aspergillosis on serum of patients with hematological disease

**DOI:** 10.1128/jcm.01053-24

**Published:** 2025-01-21

**Authors:** Hanne Lamberink, Sammy Huygens, Robina Aerts, Katrien Lagrou, Karin van Dijk, Diana Langerak, Ine Moors, Jerina Boelens, Marijke Reynders, Johan Maertens, Alexander Schauwvlieghe, Mireille van Westreenen, Ga-Lai M. Chong, Paul E. Verweij, Jochem B. Buil, Bart J. A. Rijnders

**Affiliations:** 1 Department of Internal Medicine, Section of Infectious Diseases, Erasmus University Medical Center, Rotterdam, the Netherlands; 2 Department of Medical Microbiology and Infectious Diseases, Erasmus University Medical Center, Rotterdam, the Netherlands; 3 Department of Hematology, Ghent University Hospital 60200 , Ghent, Belgium; 4 Department of Hematology, Leuven University Hospitals 60182 , Leuven, Belgium; 5 Department of Microbiology, Immunology and Transplantation, KU Leuven 573654 , Leuven, Belgium; 6 Department of Laboratory Medicine, University Hospitals Leuven 60182 , Leuven, Belgium; 7 National Reference Center for Mycosis, University Hospitals Leuven 60182 , Leuven, Belgium; 8 Department of Medical Microbiology and Infection Prevention, Amsterdam University Medical Centers 522567 , Amsterdam, the Netherlands; 9 Department of Laboratory Medicine, Ghent University Hospital 60200 , Ghent, Belgium; 10 Department of Diagnostic Sciences, Ghent University 26656 , Ghent, Belgium; 11 Department of Laboratory Medicine, Sint-Jan Brugge-Oostende General Hospital 60208 , Bruges, Belgium; 12 Department of Medical Microbiology, Sint-Jan Brugge-Oostende General Hospital 60208 , Bruges, Belgium; 13 Department of Hematology, Sint-Jan Brugge-Oostende General Hospital 60208 , Bruges, Belgium; 14 Department of Medical Microbiology, Radboud University Medical Center 6034 , Nijmegen, the Netherlands; 15 Center of Expertise for Mycology, Radboud UMC-CWZ, Nijmegen, the Netherlands; University of Calgary, Calgary, Alberta, Canada

**Keywords:** galactomannan, chemiluminescence assay, invasive aspergillosis

## Abstract

An accurate diagnosis of invasive aspergillosis (IA) in patients with underlying hematological malignancies relies heavily on galactomannan detection. In this study, we compared the VirCLIA chemiluminescence immunoassay (CLIA) with the frequently used Platelia enzyme-linked immunosorbent assay (ELISA) on serum from hematology patients with suspected IA. Patients were categorized according to EORTC/MSGERC 2020 definitions into proven/probable IA and possible/no IA. The first cohort included 161 patients at four centers, and the VirCLIA manufacturer’s cutoff of 0.200 was evaluated. Next, the optimal cutoff was determined using the Youden’s index. In a second independent cohort of 189 patients from four centers, this optimal cutoff was evaluated again. In the first cohort, sensitivities and specificities for probable/proven IA were 21.1% and 100.0% for ELISA (1.0 cutoff) and 36.6% and 95.6% (0.5 cutoff), compared to 11.3% and 97.8% for CLIA (0.200 cutoff). In the second cohort, the sensitivities of ELISA and CLIA were comparable (ELISA ≥ 1.0: 33.3%, CLIA ≥ 0.200: 38.1%). The area under the ROC curve was lower for CLIA than for ELISA in the first cohort (65.0% vs 78.7%, *P* = 0.005) but comparable in the second cohort (79.5% vs 81.3%, *P* = 0.649). Youden’s index identified 0.100 as the optimal CLIA cutoff with sensitivities of 35.2% and 61.9% in cohorts 1 and 2, respectively, at slightly reduced specificities of 85.6% and 90.5%. While the sensitivity of both assays was low to moderate at best, in patients with a high pre-test probability, we suggest 0.100 as the cutoff for the VirCLIA assay.

**IMPORTANCE:**

This study demonstrates a comparable performance of the novel chemiluminescence immunoassay (CLIA) and the conventionally used enzyme-linked immunosorbent assay for galactomannan serum testing in hematological patients at high risk for invasive aspergillosis. In patients with a high pre-test probability, a lower CLIA cutoff of 0.100 is preferred.

## INTRODUCTION

Invasive aspergillosis (IA) is a significant cause of morbidity and mortality worldwide with an annual crude mortality rate of 1.8 million deaths (1). Although it is known that prompt diagnosis and initiation of antifungal therapy lead to higher survival rates, the sensitivity of the available diagnostics on serum is usually no better than moderate (2–6). While the diagnostic armamentarium improved with the introduction of galactomannan (GM) testing on bronchoalveolar lavage (BAL) fluid, this requires a bronchoscopy. An alternative, although less sensitive, is GM testing on serum. Moreover, serum GM is correlated with clinical outcomes (7–9), and bi-weekly screening is applied within a preemptive therapeutic approach (10, 11). A frequently used GM test is the Platelia enzyme-linked immunosorbent assay (ELISA) (Bio-Rad, Marnes-la-Coquette, France). In this sandwich assay, the rat monoclonal antibody EB-A2 binds to an epitope on the side chain of the GM molecule, eventually resulting in a quantitative optical density index (ODI) (12). It is typically performed in batches, and therefore, rarely on a daily basis. Recently, Vircell (Granada, Spain) introduced a novel automated chemiluminescence immunoassay (CLIA), the *Aspergillus* Galactomannan Ag VirCLIA Monotest, to be used on individual samples, including serum and BAL samples. This assay also utilizes peroxidase-labeled rat monoclonal antibodies; however, the addition of a chemiluminescent substrate generates a glow-type radiance, quantified with relative light units (13). We previously showed a comparable sensitivity and specificity of the VirCLIA and Platelia tests on BAL fluid of hematological patients (14). In this study, we compared the Platelia ELISA and VirCLIA CLIA tests on serum of patients with underlying hematological diseases and suspected IA.

## MATERIALS AND METHODS

### Study design and patient population

The primary objective was to assess the diagnostic performance of the VirCLIA assay on serum, using the manufacturer’s cutoff of ≥0.200, in comparison to the EORTC/MSGERC 2020 definition of probable/proven IA (see below), and to evaluate other potential cutoffs. To validate these findings, the analysis was repeated in an independent cohort. The two independent cohorts were established from reference hematology centers in the Netherlands and Belgium. High-risk adult patients with hematological malignancies, either with clinical suspicion of invasive fungal disease or undergoing serum GM screening per local protocol, were included. At the time of serum collection, all patients underwent a CT scan of the lungs, either for clinical indications (e.g., fever unresponsive to antibiotics) or following a positive screening serum Platelia GM test. Only one serum sample per patient was analyzed, chosen as the sample closest to the date of the CT scan or, when applicable, to BAL sampling. In five centers, a diagnostic-driven antifungal therapy strategy was employed rather than an empirical approach, while mold-active prophylaxis was administered during remission induction therapy for AML in one center and in patients with grade II or higher GVHD in five of the six centers.

Samples for cohort 1 were prospectively collected between January 2017 and February 2023, across four centers as part of two studies (NCT03121235 and NL62004.078.17). Since patients were included prospectively, the prevalence of IA in this cohort was not predetermined. Cohort 2 served as a validation cohort and included new patients from two centers in cohort 1, as well as additional sera collected between November 2016 and September 2023 from two other hematology centers. At Leuven University Hospitals, samples were prospectively collected as part of a biobank study (S61797), whereas other centers collected and stored serum samples at −70°C during routine patient care. To simulate a 33% pre-test probability in cohort 2, two controls (i.e., patients with hematological malignancies classified as possible or no IA) were included for each IA case. Further details on informed consent and ethical approval processes are provided in the supplemental material.

### Classification of IA

BAL was performed according to the local standards, and various mycological tests were carried out on the BAL fluid obtained as part of routine care: direct microscopy with either Blankophor fluorescent whitener (Bayer AG, Leverkusen, Germany) or Calcofluor White Stain (Sigma-Aldrich, Merck, Germany), fungal culture, ELISA GM antigen testing, and *Aspergillus* DNA detection by polymerase chain reaction (PCR). For PCR, two methods were used: either an in-house PCR that targets the 28S rRNA or the commercially available CE-IVD AsperGenius 1.0 (PathoNostics, Maastricht, the Netherlands). All patients were classified according to the EORTC/MSGERC 2020 consensus definitions as cases (proven or probable IA) and controls (possible or no IA) (15).

### Validation of manufacturer’s cutoff

Both VirCLIA and Platelia tests on serum were performed centrally, and these results were used for the analysis. According to the manufacturer’s instructions, VirCLIA tests were performed using the automated chemiluminescence system from Vircell. Results comprise the ratio between the relative light units in the sample and the calibrator, which is displayed as a concentration index (CI). The manufacturer defined CI < 0.160 as negative, between 0.160 and 0.200 as equivocal, and ≥0.200 as positive (13). The CLIA results with different cutoffs of 0.160 and 0.200 were compared to the ELISA results at the cutoffs of ODI 0.5 and 1.0, the manufacturer’s cutoff as typically used in clinical practice and the cutoff as used in the EORTC/MSGERC 2020 criteria, respectively. Quantitative correlations between ELISA and CLIA for BAL and serum were explored. Sensitivity, specificity, and positive and negative predictive values were evaluated for the different cutoffs with respect to the EORTC/MSGERC 2020 definitions, and cases with discrepant results were evaluated. In a secondary analysis, the results for GM ELISA on serum and BAL fluid were excluded from the consensus definitions to avoid incorporation bias, which would ultimately favor the ELISA.

### Findings and validating the optimal cutoff

With the findings from the first cohort, the optimal cutoff for CLIA was estimated using the Youden index (YI). This optimal cutoff was compared to different cutoffs of the ELISA and subsequently evaluated again in the second cohort.

### Evaluating the reproducibility of CLIA on serum

Eleven serum samples from the first cohort were tested sequentially to assess the precision and reproducibility of the CLIA. All tests were conducted by the same technician in the same laboratory every few days, ensuring consistent conditions by controlling the number of freeze/thaw cycles. Variability was evaluated by calculating the coefficient of variation for each sample, determined by dividing the standard deviation by the mean.

### Statistics

After calculating sensitivity, specificity, and positive and negative predictive values, McNemar’s test for paired proportions was used to compare the diagnostic performance of ELISA and CLIA. Receiver operating characteristic (ROC) curves were obtained to calculate the area under the curve (AUC), and Youden’s *J*-statistic was calculated using the coordinates of the ROC curves. The overall agreement between ELISA and CLIA was calculated, together with Cohen’s Kappa coefficient (*κ*) for qualitative agreement. Quantitative agreement was assessed using Spearman’s rho (*ρ*) for nonparametric data. Details of the interpretation of the results can be found in the supplemental material. All analyses were performed with SPSS version 28.0.1 and R version 4.3.3.

## RESULTS

### Population

The first cohort consisted of 161 patients, and the second cohort consisted of 189 patients; see Table 1 for the baseline characteristics and all mycological test results. The proportion of patients with neutropenia (neutrophil count < 0.5 × 10^9^/L), who had undergone allogeneic stem cell transplantation or received anti-mold therapy or prophylaxis at the time of serum sampling, was comparable between the two cohorts. According to the EORTC/MSGERC 2020 consensus definitions, proven or probable IA was present in 71/161 (44.1%) patients in cohort 1, and in 63/189 (33.3%) patients in cohort 2 in accordance with the previously defined case-control ratio of 1:2. Therefore, both cohorts represent a very high-risk population with a relatively high rate of true cases (proven or probable IA) compared with controls (possible or no IA).

**TABLE 1:** Baseline characteristics for all patients and for cohort 1 (proof of concept) and cohort 2 (validation cohort) separately^*f*^^,^^*g*^

	All patients (*n* = 350) median (IQR) or *n* (%)	Cohort 1 (*n* = 161) median (IQR) or *n* (%)	Cohort 2 (*n* = 189) median (IQR) or *n* (%)
Age (years)	61.0 (51.8–68.0)	63.0 (55.5–70.0)	60.0 (50.0–67.0)
Sex (male)	221 (63.1)	108 (67.1)	113 (59.8)
Underlying hematological malignancy	350 (100.0)	161 (100.0)	189 (100.0)
AML	174 (49.7)	77 (47.8)	97 (51.3)
MDS	38 (10.9)	15 (9.3)	23 (12.2)
ALL	26 (7.4)	11 (6.8)	15 (7.9)
Others	112 (32.0)	58 (36.0)	54 (28.6)
HSCT	345 (98.6)	156 (96.9)	189 (100.0)
Allogeneic	110 (31.9)	43 (27.6)	67 (35.4)
Autologous	13 (3.8)	7 (4.5)	6 (3.2)
None	222 (64.3)	106 (67.9)	116 (61.4)
Neutropenia^*a*^	158/291 (54.3)	51/102 (50.0)	107/189 (56.6)
No antifungal therapy^*b*^	123/286 (43.0)	34/97 (35.1)	89/189 (47.1)
Fluconazole	70 (24.5)	37 (38.1)	33 (17.5)
Mold-active antifungal therapy or prophylaxis	93 (32.5)	26/97 (26.8)	67/189 (35.5)
Voriconazole	33 (11.5)	13 (13.4)	20 (10.6)
Posaconazole	11 (3.8)	3 (3.1)	8 (4.2)
Isavuconazole	12 (4.2)	0 (0)	12 (6.3)
Echinocandins	5 (1.7)	2 (2.1)	3 (1.6)
L-AmB	14 (4.9)	2 (2.1)	12 (6.3)
Combination therapy	15 (5.2)	6 (6.2)	9 (4.8)
Others	3 (1.0)	0 (0)	3 (1.6)
Fungal diagnostics on BAL			
Microscopy positivity^*c*^	19/220 (8.6)	9/129 (7.0)	10/91 (11.0)
Fungal culture positivity for *Aspergillus* species	29/242 (12.0)	17/150 (11.3)	12/92 (13.0)
*Aspergillus fumigatus*	28/242 (8.0)	16/150 (9.9)	12/92 (6.3)
*Aspergillus niger*	1/242 (0.3)	1/150 (0.6)	0/92 (0)
GM positivity (ELISA, ≥1.0)	77/216 (35.6)	44/146 (30.1)	33/70 (47.1)
GM ODI (ELISA)	0.40 (0.10–2.38)	0.30 (0.10–2.00)	0.64 (0.10–3.50)
GM positivity (CLIA, ≥0.200)	24/63 (38.1)	17/41 (41.5)	7/22 (31.8)
GM CI (CLIA)	0.09 (0.04–0.98)	0.10 (0.02–1.10)	0.08 (0.06–0.88)
*Aspergillus* PCR positivity (in duplicate)	74/212 (34.9)	48/133 (36.1)	26/79 (32.9)
GM positivity on serum (ELISA, ≥0.5)^*d*^	25/260 (9.6)	10/71 (14.1)	15/189 (7.9)
GM ODI on serum (ELISA)	0.07 (0.04–0.10)	0.1 (0–0.2)	0.06 (0.04–0.1)
EORTC/MSGERC criteria^*e*^	350 (100.0)	161 (100.0)	189 (100.0)
Proven IA	11 (3.1)	6 (3.7)	5 (2.6)
Probable IA	123 (35.1)	65 (40.4)	58 (30.7)
Possible IA	99 (28.3)	68 (42.2)	31 (16.4)
No IA/unclassifiable	117 (33.4)	22 (13.7)	95 (50.3)

^*a*^ ≤0.5 × 10^9^/L at the time of serum sampling (±48 hours).

^*b*^ In the last 24 hours before serum sampling.

^*c*^ Microscopic detection of fungal elements in BAL fluid indicating a mold.

^*d*^ Serum GM positivity based on results from local laboratory when available.

^*e*^ The EORTC/MSGERC 2020 consensus definitions were used to classify patients.

^*f*^ ALL, acute lymphoblastic leukemia; AML, acute myeloid leukemia; BAL, bronchoalveolar lavage; CI, concentration index; CLIA, chemiluminescence immunoassay (VirCLIA Galactomannan AG assay); EIA, ELISA-based galactomannan testing (Platelia); EORTC/MSGERC, European Organization for Research and Treatment of Cancer and the Mycoses Study Group Education and Research Consortium; HSCT, hematopoietic stem cell transplantation; IA, invasive aspergillosis; L-AmB, liposomal amphotericin B; MDS, myelodysplastic syndrome; and ODI, optical density index.

^*g*^ If information was missing, we reported the denominator for which the data were known within the cohort.

### Sensitivity and specificity of serum ELISA

As previously stated, serum samples were collected either near the time of chest CT or BAL when there was clinical suspicion of a fungal infection or following a positive serum Platelia result when part of a screening protocol. The serum GM results of ELISA and CLIA at various cutoff values were compared to the EORTC/MSGERC 2020 consensus definitions (Table 2). ELISA results were positive (ODI ≥ 1.0) in 15/161 (9.3%) patients in cohort 1 (sensitivity, 21.1%; specificity, 100.0% for probable or proven IA). In cohort 2, this was the case in 22/189 (11.6%) patients (sensitivity 33.3%, specificity 99.2%). With an ODI ≥ 0.5 as the cutoff, sensitivity increased to 36.6% and 49.2% for cohorts 1 and 2, respectively, with a small loss in specificity (>95% for both cohorts). Additional analyses were performed to correct for ELISA GM incorporation bias by excluding results from serum and BAL ELISA for the classification of cases, which did not change the conclusions (Table 2). An overview of the quantitative correlations between serum ELISA and both BAL ELISA and CLIA is provided in Table S2.

**TABLE 2:** Performance of the ELISA (Platelia) and CLIA (VirCLIA) galactomannan assays on serum as compared to the EORTC/MSGERC 2020 consensus definitions for the two cohorts^*i*^^,^^*j*^

	Sensitivity cohort 1 (%)	Sensitivity cohort 2 (%)	Diff.	Specificity cohort 1 (%)	Specificity cohort 2 (%)	Diff.	PPV cohort 1 (%)	PPV cohort 2 (%)	Diff.	NPV cohort 1 (%)	NPV cohort 2 (%)	Diff.	ROC AUC cohort 1 (%, 95%CI)	ROC AUC cohort 2 (%, 95%CI)
Proven/probable vs possible/no IA														
GM ELISA ≥ 1.0	21.1	33.3	12.2	100.0	99.2	−0.8	100.0	95.5	−4.5	61.6	74.9	13.3	ELISA: 78.7 (71.4–85.9)	ELISA: 81.3 (74.3–88.3)
GM ELISA ≥ 0.5	36.6	49.2^*c*^	12.6	95.6	96.8^*f*^	1.2	86.7	88.6	1.9	65.7	79.2	13.5		
GM CLIA ≥ 0.2	11.3	38.1	26.8	97.8	99.2	1.4	80.0	96.0	16.0	58.3	76.2	17.9	CLIA: 65.0 (56.4–73.6)	CLIA: 79.5 (72.3–86.8)
GM CLIA ≥ 0.16	16.9	47.6	30.7	97.8	96.8	−1.0	85.7	88.2	2.5	59.9	78.7	18.8		
GM CLIA ≥ 0.10	35.2	61.9^*c*^	26.7	85.6	90.5^*f*^	4.9	65.8	76.5	10.7	62.6	82.6	20.0		
Proven/probable vs possible/no IA (serum GM excluded)^*a*^														
GM ELISA ≥ 1.0	16.4	30.0	13.6	95.7	95.0	−0.7	73.3	68.2	−5.1	61.6	79.0	17.4	ELISA: 74.5 (66.5–82.4)	ELISA: 75.7 (67.2–84.1)
GM ELISA ≥ 0.5	32.8	46.0^*d*^	13.2	91.5	91.4^*g*^	−0.1	73.3	65.7	−7.6	65.7	82.5	16.8		
GM CLIA ≥ 0.2	11.9	34.0	22.1	97.9	94.2	−3.7	80.0	68.0	−12.0	60.9	79.9	19.0	CLIA: 63.4 (54.6–72.2)	CLIA: 77.0 (68.7–85.3)
GM CLIA ≥ 0.16	16.4	46.0	29.6	96.8	92.1	−4.7	78.6	67.7	−10.9	61.9	82.6	20.7		
GM CLIA ≥ 0.10	34.3	62.0^*d*^	27.7	84.0	85.6^*g*^	1.6	60.5	60.8	0.3	64.2	86.2	22.0		
Proven/probable vs possible/no IA (GM excluded)^*b*^														
GM ELISA ≥ 1.0	15.5	34.4	18.9	94.2	93.0	−1.2	60.0	50.0	−10.0	66.4	87.4	21.0	ELISA: 70.1 (61.4–78.8)	ELISA: 75.4 (65.1–85.6)
GM ELISA ≥ 0.5	29.3	50.0^*e*^	20.7	87.4	87.9^*h*^	0.5	56.7	45.7	−11.0	68.7	89.6	20.9		
GM CLIA ≥ 0.2	12.1	34.4	22.3	97.1	91.1	−6.0	70.0	44.0	−26.0	66.2	87.2	21.0	CLIA: 57.6 (48.3–66.9)	CLIA: 82.6 (73.5–91.8)
GM CLIA ≥ 0.16	15.5	50.0	34.5	95.1	88.5	−6.6	64.3	47.1	−17.2	66.7	89.7	23.0		
GM CLIA ≥ 0.10	31.0	71.9^*e*^	40.9	80.6	82.2^*h*^	1.6	47.4	45.1	−2.3	67.5	93.5	26.0		

^*a*^ Secondary analysis performed in which serum ELISA GM was excluded from the definition of probable/proven IA to correct for possible incorporation bias.

^*b*^ Third analysis performed in which serum ELISA GM and BAL ELISA GM were excluded from the definition of probable/proven IA.

^*c*^ *P* = 0.039.

^*d*^ *P* = 0.039.

^*e*^ *P* = 0.039.

^*f*^ *P* = 0.021.

^*g*^ *P* = 0.021.

^*h*^ *P* = 0.022.

^*i*^ The column Diff. shows the difference in percentage between cohorts 2 and 1 for sensitivity, specificity, and PPV and NPV.

^*j*^ AUC, area under the curve; CI, confidence interval; CLIA, chemiluminescence immunoassay (VirCLIA Galactomannan AG assay); Diff., difference; ELISA, ELISA-based galactomannan testing (Platelia); GM, galactomannan; IA, invasive aspergillosis; NPV, negative predictive value; PPV, positive predictive value; and ROC, receiver operating characteristic.

### Sensitivity and specificity of serum CLIA

**Fig 1:**
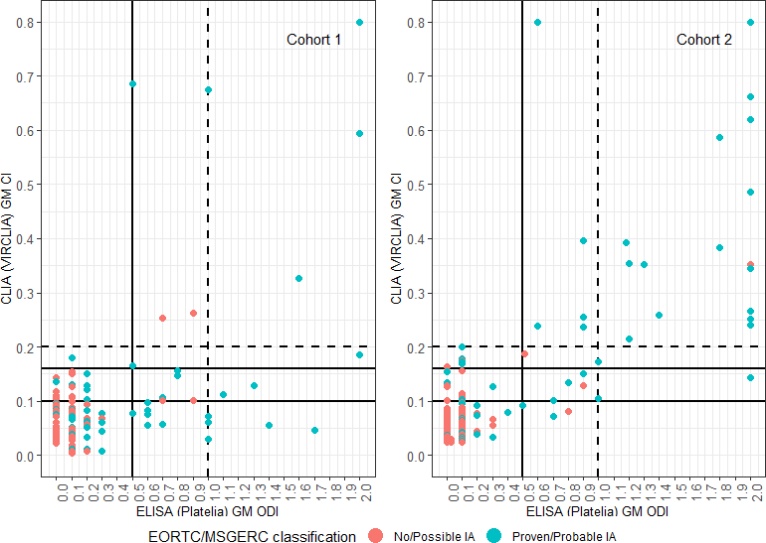
Detailed overview of the comparison between CLIA GM CI and ELISA GM ODI values for 161 serum samples in cohort 1 and 189 serum samples in cohort 2 from patients with hematological malignancies and suspected invasive aspergillosis. Patients were classified in accordance with the EORTC/MSGERC 2020 criteria. Very high values are displayed at a maximum of *x* = 2.0 or *y* = 0.8 for clarity. CI, concentration index; CLIA, chemiluminescence immunoassay (VirCLIA Galactomannan AG assay); ELISA, ELISA-based galactomannan testing (Platelia); GM, galactomannan; IA, invasive aspergillosis; and ODI, optical density index.

Serum CLIA was positive at the manufacturer’s cutoff CI ≥ 0.200 in 10/161 (6.2%) samples in cohort 1, with a sensitivity of 11.3% and a specificity of 97.8% for probable or proven IA. Serum CLIA was positive (CI ≥ 0.200) in 25/189 (13.2%) samples in cohort 2 with a sensitivity of 38.1% and a specificity of 99.2%. Serum CLIA was positive (CI ≥ 0.160) in 14/161 (8.7%) samples in cohort 1 (sensitivity 16.9% and specificity 97.8%) and in 34/189 (18.0%) samples in cohort 2 (sensitivity 47.6% and specificity 96.8%), see Table 2. Additional analyses showed no relevant influence of incorporation bias. Figure 1 displays the absolute values of the two assays for the two cohorts separately for cases and controls. An overview of the quantitative correlations between serum CLIA and both BAL ELISA and CLIA is provided in Table S2.

### Optimal cutoff values for serum ELISA and CLIA assays

In cohort 1, at the cutoff of ≥0.200, CLIA had a lower sensitivity of 11.3% compared to 21.1% with ELISA at a cutoff of ≥1.0 (*P* = 0.039), but this difference was not observed in cohort 2 (sensitivity 33.3% and 38.1%, respectively, *P* = 0.508). At a lower cutoff of 0.100, the sensitivity of CLIA was 35.2%, which was higher than that of ELISA (≥1.0) at 21.1% in cohort 1 (*P* = 0.041) and cohort 2 (61.9% versus 33.3%, *P* < 0.001). The sensitivity of the 0.100 CLIA cutoff was still higher than that of the 0.5 ELISA cutoff in cohort 2, although less pronounced. As expected, the increase in sensitivity at the 0.100 cutoff resulted in some loss in specificity (Table 2). That a cutoff of close to 0.100 is probably the best compromise between sensitivity and specificity was confirmed with YI analyses on both cohorts. According to the YI, the best cutoff values in cohort 1 were 0.050 (YI, 0.240; sensitivity, 66.2%; and specificity, 57.8%) and 0.091 (YI, 0.563; sensitivity, 66.7%; specificity, 89.7%) in cohort 2. Despite the higher sensitivity of the 0.05 cutoff in cohort 1, the very low specificity of this cutoff is not acceptable in clinical practice.

### Concordance of serum ELISA and CLIA assays

The qualitative agreement between ELISA and CLIA was assessed for different cutoff values. Table 3 shows a high overall agreement for the manufacturers’ cutoffs (ELISA 0.5 and CLIA 0.200): 87.6% in cohort 1 and 93.7% in cohort 2 with Cohen’s kappa indicating moderate and substantial qualitative agreement, respectively. The quantitative agreement for ELISA and CLIA was weak for cohort 1 (Spearman’s *ρ* 0.298 [95%CI 0.145–0.436], *P* < 0.001) and moderate for cohort 2 (Spearman’s *ρ* 0.467 [95%CI 0.344–0.574], *P* < 0.001). The discriminative power of CLIA was lower than that of ELISA in cohort 1 (see Fig. S1). This difference was not observed in cohort 2.

**TABLE 3:** Qualitative agreement between ELISA and CLIA galactomannan assays on serum for cohorts 1 and 2^*a*^^,^^*b*^

CLIA CI cutoff value	Agreement (% [Cohen’s *κ*]) with ELISA ODI cutoff value of:	
	≥1.0	≥0.5
Cohort 1		
≥0.200	93.2 (0.525)	87.6 (0.449)
≥0.160	91.9 (0.507)	87.6 (0.484)
≥0.100	79.5 (0.281)	81.4 (0.443)
Cohort 2		
≥0.200	95.2 (0.781)	93.7 (0.764)
≥0.160	91.5 (0.667)	91.0 (0.699)
≥0.100	84.7 (0.526)	88.4 (0.672)

^*a*^ The manufacturer’s cutoffs for positivity are ODI 0.5 for ELISA (Platelia) and CI 0.200 for CLIA (VirCLIA).

^*b*^ CI, concentration index; CLIA, chemiluminescence immunoassay (VirCLIA Galactomannan AG assay); ELISA, ELISA-based galactomannan testing (Platelia); GM, galactomannan; and ODI, optical density index.

### Evaluation of discrepant serum ELISA and CLIA assay results

From all 350 patients, discrepant results for CLIA (0.200) and ELISA (1.0) were observed in 20 cases. Nine cases had positive CLIA and negative ELISA results, and 11 had negative CLIA and positive ELISA results. The details are provided in Table S1A. Furthermore, an overview of 20 additional cases with discrepant results between CLIA (0.200) and ELISA (0.5) and 37 additional cases with discrepant results between CLIA (0.100) and ELISA (0.5) is provided in Table S1B and C.

### Reproducibility

Eleven serum samples from the first cohort were sequentially tested to evaluate the precision and reproducibility of CLIA. Five serum samples showed a relatively good reproducibility: two samples with CI ≥ 0.200 (coefficient of variation [CV] 13.0%–19.7%) and three samples with CI < 0.100 (CV 28.7%–30.5%), see Table S3. However, six sera with an initial result between 0.100 and 0.200 showed less precision, with a CV ranging from 26.0% to 121.0%, possibly changing the interpretation of the test results from negative to positive or vice versa.

## DISCUSSION

### Main findings

In this comparative study of the Platelia and VirCLIA galactomannan assays in a population with hematological malignancies and a high pretest probability of IA, the sensitivity and specificity were lower for CLIA at the cutoff of ≥0.200 than for ELISA (cutoff ≥ 1.0) in cohort 1 but comparable in cohort 2. Notably, when the cutoff value of CLIA was lowered to 0.100, the sensitivity of CLIA increased substantially, with only a relatively small loss of specificity. At this 0.100 CLIA cutoff, its sensitivity was at least as good as that of ELISA at the lower cutoff of 0.5, which is frequently used in clinical practice. We suggest that in populations with a high pretest probability, meaning at the time of clinical suspicion of IA in a high-risk host, 0.100 should be used as a cutoff in order to avoid missing an IA infection as much as possible. Given that the reproducibility of a weakly positive serum GM test is not perfect, we recommend confirmation of a positive GM test result on a new serum sample, especially when the CLIA CI is between 0.100 and 0.200.

### Comparison with previous studies

These findings differ from those of Calero et al. (16), who reported significantly higher sensitivity and specificity for CLIA compared to ELISA with regard to the EORTC/MSGERC criteria: 80.9% and 100% vs 63.2% and 100%, respectively. These differences may stem from the pooling of data from both serum and bronchoalveolar lavage fluid samples in their study, whereas our study focused solely on serum samples. Also, differences in patient characteristics (e.g., use of antifungal prophylaxis or therapy at the time of serum sampling in a substantial proportion of our patient population) as well as the application of a test as a screening tool (e.g., twice a week) rather than a diagnostic test at the time of suspected infection can impact the results. However, these details were not provided in the article, limiting direct comparisons.

### Strengths and limitations

The strength of this study is the use of two independent cohorts to confirm the findings in cohort 1: in particular, the test performance at the 0.100 cutoff. The differences in sensitivity and specificity observed in both cohorts were surprising and could be a coincidental finding. However, it may also be partially explained by the actual differences between the two populations. For instance, all serum samples in cohort 1 came from patients included in a study (NCT03121235) that only enrolled patients with lung CT findings suggestive of invasive fungal disease. In cohort 2, this was not the case, so any lung abnormality was allowed. The relatively low sensitivities we observed are probably the consequence of a protocol that results in the performance of lung CT after >72–96 hours of persistent fever, which is typically followed by a BAL when intrapulmonary abnormalities are present. This results in an earlier diagnosis of IA compared with studies on the ELISA GM assay performed many years or decades ago when this lung CT-guided policy was less frequent. Despite its limitations, our study benefits from the inclusion of two large cohorts from large hematology reference centers in Belgium and the Netherlands. This enhances the generalizability and reliability of our findings.

In conclusion, our study demonstrates a comparable performance of CLIA and ELISA for GM serum testing in hematological patients at high risk for invasive aspergillosis. In patients with a high pre-test probability, a lower CLIA cutoff of 0.100 is preferred.

## SUPPLEMENTAL MATERIAL

Supplemental material jcm.01053-24-s0001.docx Supplemental text, Tables S1 to S3, and Fig. S1.

ASM does not own the copyrights to Supplemental Material that may be linked to, or accessed through, an article. The authors have granted ASM a non-exclusive, world-wide license to publish the Supplemental Material files. Please contact the corresponding author directly for reuse.
