# Correction for Lamberink et al., “Multicenter validation of a galactomannan chemiluminescence immunoassay for the diagnosis of pulmonary aspergillosis on serum of patients with hematological disease”

**DOI:** 10.1128/jcm.00394-25

**Published:** 2025-03-25

**Authors:** Hanne Lamberink, Sammy Huygens, Robina Aerts, Katrien Lagrou, Karin van Dijk, Diana Langerak, Ine Moors, Jerina Boelens, Marijke Reynders, Johan Maertens, Alexander Schauwvlieghe, Mireille van Westreenen, Ga-Lai M. Chong, Paul E. Verweij, Jochem B. Buil, Bart J. A. Rijnders

## AUTHOR CORRECTION

Volume 63, issue 2, e01053-24, https://doi.org/10.1128/jcm.01053-24. Supplemental material: The supplemental file has been replaced, as the previous version was corrupted and could not be opened after download. The contents of the supplemental file have not been changed and are provided in this correction.

## SUPPLEMENTAL MATERIAL

Supplemental material jcm.00394-25-s0001.docx Supplemental text, Tables S1 to S3, and Fig. S1.

ASM does not own the copyrights to Supplemental Material that may be linked to, or accessed through, an article. The authors have granted ASM a non-exclusive, world-wide license to publish the Supplemental Material files. Please contact the corresponding author directly for reuse.

