# Supplementary material for: Correction for Lamberink et al., “Multicenter validation of a galactomannan chemiluminescence immunoassay for the diagnosis of pulmonary aspergillosis on serum of patients with hematological disease”
Source: J Clin Microbiol. 2025 Mar 25;63(5):e00394-25. doi: 10.1128/jcm.00394-25 (PMC12077167; doi:10.1128/jcm.00394-25)
Supplement: Supplemental material — Supplemental text, Tables S1 to S3, and Fig. S1. [file jcm.00394-25-s0001.docx]

**Supplementary materials**

**Statistical analyses**

Receiver operating characteristic (ROC) curves were obtained for calculation of the area under the curve (AUC) where an AUC <0.50 represents no discrimination, 0.50-0.69 poor discrimination, 0.70-0.79 acceptable discrimination, 0.80-0.89 excellent discrimination and ≥0.90 outstanding discrimination^1^. Cohen’s Kappa coefficient (κ) was obtained in which 0.41-0.60 represents moderate agreement, 0.61-0.80 substantial agreement and 0.81-1.00 almost perfect agreement^2^. The quantitative agreement was assessed with Spearman’s rho (ρ) for non-parametric data in which 0.21-0.40 represents weak agreement, 0.41-0.60 moderate agreement, 0.61-0.80 strong agreement and 0.81-1.00 very strong agreement^3^.

**References**

1. Hosmer Jr DW, Lemeshow S, Sturdivant RX. Applied logistic regression: John Wiley & Sons, 2013.

2. Landis JR, Koch GG. The measurement of observer agreement for categorical data. Biometrics 1977; 33: 159-74.

3. Prion S, Haerling KA. Making Sense of Methods and Measurement: Spearman-Rho Ranked-Order Correlation Coefficient. Clinical Simulation in Nursing 2014; 10: 535-6.

**Supplementary Table 1A. Details of 20 cases in which discrepant results were found between serum ELISA (ODI ≥1.0) and serum CLIA (CI <0.200) and serum ELISA (ODI<1.0) and serum CLIA (CI ≥0.200). Note that in a few patients, the probable or proven diagnosis was based on an on-site galactomannan result from another serum sample than the one that was tested in the central lab. Therefore, a few patients were classified as probable despite the absence of a positive mycological test in the table.**

Abbreviations: ALL = acute lymphoblastic leukemia; AML = acute myeloid leukemia; BAL = bronchoalveolar lavage; CI = concentration index; CLL = chronic lymphocytic leukemia; DLBCL = diffuse large B cell lymphoma; GM = galactomannan; HLH = hemophagocytic lymphohistiocytosis; IA = invasive aspergillosis; MDS = myelodysplastic syndrome; MM = multiple myeloma; NK/T cell lymphoma = natural killer/T cell lymphoma; NA = not available; ODI = optical density index; PCR = polymerase chain reaction; PNH = paroxysmal nocturnal hemoglobinuria.

|  | | | | | **BAL fluid results** | | | |  |
| --- | --- | --- | --- | --- | --- | --- | --- | --- | --- |
| **Patient no.** | **Underlying disease** | **Radiological finding(s)** | **Serum ELISA ODI** | **Serum CLIA CI** | **Direct microscopy** | **Fungal culture** | **ELISA ODI** | ***Aspergillus* PCR** | **EORTC/MSGERC 2020 classification (excluding GM)** |
| **1** | DLBCL | Halo sign | 9.5 | 0.143 | Negative | *A. fumigatus* | 9.8 | Positive in duplicate | Probable IA |
| **2** | MDS | Halo sign | 2.2 | 0.185 | NA | NA | NA | NA | Probable IA (possible IA) |
| **3** | AML | Nodules | 1.7 | 0.047 | NA | NA | NA | NA | Probable IA (possible IA) |
| **4** | NK/T cell lymphoma | Halo sign | 1.4 | 0.056 | Negative | Negative | 0.1 | Species negative | Probable IA (possible IA) |
| **5** | ALL | Nodule | 1.3 | 0.128 | Negative | Negative | 5.3 | Positive in duplicate | Probable IA |
| **6** | AML | Halo sign | 1.1 | 0.113 | Negative | Negative | 1.00 | Species negative | Probable IA (possible IA) |
| **7** | MM | Nodules | 1.0 | 0.172 | Negative | *A. fumigatus* | 8.9 | Positive in duplicate | Probable IA |
| **8** | AML | Nodules | 1.0 | 0.105 | Negative | Negative | NA | Species negative | Probable IA (possible IA) |
| **9** | AML | Nodules | 1.0 | 0.072 | Negative | Negative | 5.0 | NA | Probable IA (possible IA) |
| **10** | MM | Halo sign | 1.0 | 0.061 | NA | NA | NA | Species negative | Proven IA |
| **11** | PNH | Nodules, cavities | 1.0 | 0.030 | Negative | *A. niger* | 0.2 | Species negative | Probable IA |
| **12** | AML | Halo sign | 0.9 | 0.396 | Negative | *A. fumigatus* | 8.5 | Positive in duplicate | Probable IA |
| **13** | Aplastic anemia | Nodules | 0.9 | 0.263 | NA | NA | NA | NA | Possible IA |
| **14** | DLBCL | Halo sign, nodules, cavity | 0.9 | 0.255 | NA | NA | NA | NA | Probable IA (possible IA) |
| **15** | AML | Halo sign | 0.9 | 0.236 | Negative | Negative | 0.3 | Species positive (single) | Probable IA (possible IA) |
| **16** | CLL | Tree in bud, ground-glass (atypical) | 0.7 | 0.254 | Negative | Negative | NA | Species positive (single) | Unclassifiable |
| **17** | AML | Nodules, cavities | 0.6 | 1.005 | Negative | Negative | 12.2 | Species positive (single) | Probable IA (possible IA) |
| **18** | ALL | Nodules | 0.6 | 0.238 | Negative | Negative | 0.4 | Positive in duplicate | Probable IA |
| **19** | HLH | Halo sign | 0.5 | 0.686 | Negative | Negative | 5.6 | Positive in duplicate | Probable IA |
| **20** | AML | Other infectious changes | 0.1 | 0.201 | Negative | Negative | 5.6 | Species negative | Probable IA (possible IA) |

**Supplementary Table 1A. Details of 20 cases in which discrepant results were found between serum ELISA (ODI ≥1.0) and serum CLIA (CI <0.200) and serum ELISA (ODI<1.0) and serum CLIA (CI ≥0.200). Note that in a few patients, the probable or proven diagnosis was based on an on-site galactomannan result from another serum sample than the one that was tested in the central lab. Therefore, a few patients were classified as probable despite the absence of a positive mycological test in the table.**

Abbreviations: ALL = acute lymphoblastic leukemia; AML = acute myeloid leukemia; BAL = bronchoalveolar lavage; CI = concentration index; CLL = chronic lymphocytic leukemia; DLBCL = diffuse large B cell lymphoma; GM = galactomannan; HLH = hemophagocytic lymphohistiocytosis; IA = invasive aspergillosis; MDS = myelodysplastic syndrome; MM = multiple myeloma; NK/T cell lymphoma = natural killer/T cell lymphoma; NA = not available; ODI = optical density index; PCR = polymerase chain reaction; PNH = paroxysmal nocturnal hemoglobinuria.

|  | | | | | **BAL fluid results** | | | |  |
| --- | --- | --- | --- | --- | --- | --- | --- | --- | --- |
| **Patient no.** | **Underlying disease** | **Radiological finding(s)** | **Serum ELISA ODI** | **Serum CLIA CI** | **Direct microscopy** | **Fungal culture** | **ELISA ODI** | ***Aspergillus* PCR** | **EORTC/MSGERC 2020 classification (excluding GM)** |
| 21 | DLBCL | Nodules | 0.9 | 0.150 | Negative | Negative | 4.7 | Positive in duplicate | Probable IA |
| 23 | Follicular lymphoma | Nodules, wedge-shaped consolidation | 0.9 | 0.129 | NA | NA | NA | NA | Possible IA |
| 23 | Polycythaemia vera | Nodules | 0.9 | 0.102 | NA | NA | NA | NA | Possible IA |
| 24 | DLBCL | Halo sign, wedge-shaped consolidation | 0.8 | 0.157 | Negative | Negative | 2.9 | NA | Probable IA (possible IA) |
| 25 | ALL | Halo sign | 0.8 | 0.147 | Negative | *A. fumigatus* | 5.3 | Positive in duplicate | Proven IA |
| 26 | AML | Halo sign | 0.8 | 0.135 | Negative | Negative | 1.0 | NA | Probable IA (possible IA) |
| 27 | ALL | Nodule | 0.8 | 0.081 | NA | NA | NA | NA | Possible IA |
| 28 | AML | Halo sign | 0.7 | 0.106 | Negative | Negative | 9.0 | Species negative | Probable IA |
| 29 | PMF | Consolidation, tree-in-bud (atypical) | 0.7 | 0.102 | Negative | Negative | 0.7 | Species negative | Unclassifiable |
| 30 | AML | Nodules | 0.7 | 0.102 | Negative | Negative | 3.2 | Positive in duplicate | Probable IA |
| 31 | AML | Other infectious changes | 0.7 | 0.071 | Negative | Negative | 0.7 | Species positive (single) | Probable IA (possible IA) |
| 32 | AML | Nodules | 0.7 | 0.057 | Positive (mycelia) | Negative | 7.8 | Species positive (single) | Probable IA |
| 33 | MDS | Nodules, wedge-shaped consolidations | 0.6 | 0.098 | Negative | *A. fumigatus* | 4.7 | Positive in duplicate | Probable IA |
| 34 | Primary CNS lymphoma | Halo sign | 0.6 | 0.083 | Negative | Negative | 2.9 | Positive in duplicate | Probable IA |
| 35 | AML | Halo sign | 0.6 | 0.076 | Negative | Negative | 2.7 | Positive in duplicate | Probable IA |
| 36 | MDS | Halo sign | 0.6 | 0.055 | Negative | Negative | 6.7 | Positive in duplicate | Probable IA |
| 37 | AML | Halo sign | 0.5 | 0.188 | Negative | Negative | 0.3 | NA | Possible IA |
| 38 | AML | Halo sign | 0.5 | 0.165 | Negative | Negative | 6.1 | Positive in duplicate | Probable IA |
| 39 | ALL | Halo sign | 0.5 | 0.092 | Negative | *A. fumigatus* | NA | Positive in duplicate | Proven IA |
| 40 | Accelerated phase MPN | Halo sign, cavity | 0.5 | 0.077 | Negative | Negative | 1.7 | Species negative | Probable IA (possible IA) |

**Supplementary Table 1B. Details of 20 cases in which discrepant results were found between serum ELISA (ODI ≥0.5) and serum CLIA (CI <0.200). Note that in a few patients, the probable or proven diagnosis was based on an on-site galactomannan result from another serum sample than the one that was tested in the central lab. Therefore, a few patients were classified as probable despite the absence of a positive mycological test in the table.**

Abbreviations: ALL = acute lymphoblastic leukemia; AML = acute myeloid leukemia; BAL = bronchoalveolar lavage; CI = concentration index; DLBCL = diffuse large B cell lymphoma; GM = galactomannan; IA = invasive aspergillosis; MDS = myelodysplastic syndrome; MPN = myeloproliferative neoplasm; NA = not available; ODI = optical density index; PCR = polymerase chain reaction; PMF = primary myelofibrosis.

|  | | | | | **BAL fluid results** | | | |  |
| --- | --- | --- | --- | --- | --- | --- | --- | --- | --- |
| **Patient no.** | **Underlying disease** | **Radiological finding(s)** | **Serum ELISA ODI** | **Serum CLIA CI** | **Direct microscopy** | **Fungal culture** | **ELISA ODI** | ***Aspergillus* PCR** | **EORTC/MSGERC 2020 classification (excluding GM)** |
| 41 | ALL | Halo sign | 0.3 | 0.126 | Positive | Negative | NA | Positive in duplicate | Probable IA (probable IA) |
| 42 | MCL | Nodules | 0.2 | 0.150 | Negative | *A. fumigatus* | 3.1 | Positive in duplicate | Probable IA (probable IA) |
| 43 | ALL | Halo sign, wedge-shaped consolidation | 0.2 | 0.129 | Positive | *A. fumigatus* | 8.0 | Positive in duplicate | Probable IA (probable IA) |
| 44 | AML | Halo sign | 0.2 | 0.122 | NA | Negative | 2.2 | Species negative | Probable IA (possible IA) |
| 45 | ALL | Halo sign | 0.2 | 0.103 | Negative | Negative | 3.4 | Positive in duplicate | Probable IA (probable IA) |
| 46 | NHL | Nodules | 0.1 | 0.180 | NA | Negative | 4.0 | Positive in duplicate | Probable IA (probable IA) |
| 47 | DLBCL | Cavity | 0.1 | 0.180 | NA | Negative | 3.4 | Species positive (single) | Probable IA (possible IA) |
| 48 | MDS | Other infectious changes | 0.1 | 0.178 | Negative | Negative | 3.1 | Positive in duplicate | Probable IA (probable IA) |
| 49 | AML | Nodules | 0.1 | 0.174 | Negative | Negative | 0.2 | Species negative | Possible IA (possible IA) |
| 50 | AML | Nodules | 0.1 | 0.172 | Positive | *A. fumigatus* | 5.4 | Positive in duplicate | Probable IA (probable IA) |
| 51 | MDS | Nodules | 0.1 | 0.171 | Negative | Negative | 1.9 | Species negative | Probable IA (possible IA) |
| 52 | ALL | Halo sign | 0.1 | 0.169 | Negative | Negative | 0.5 | Positive in duplicate | Probable IA (probable IA) |
| 53 | Unknown | Nodules | 0.1 | 0.169 | Positive | *A. fumigatus* | 0.4 | Species positive (single) | Proven IA |
| 54 | AML | Halo sign | 0.1 | 0.157 | NA | NA | NA | NA | Possible IA (possible IA) |
| 55 | ALL | Halo sign | 0.1 | 0.155 | Negative | Negative | 0.3 | Species negative | Possible IA (possible IA) |
| 56 | ALL | Halo sign | 0.1 | 0.150 | Negative | Negative | 0.2 | Species positive (single) | Possible IA (possible IA) |
| 57 | Angioimmunoblastic T-cell lymphoma | Halo sign, nodules | 0.1 | 0.131 | Negative | *A. fumigatus* | 1.5 | Positive in duplicate | Probable IA (probable IA) |
| 58 | AML | Halo sign | 0.1 | 0.128 | NA | Negative | 0.8 | Positive in duplicate | Probable IA (probable IA) |
| 59 | MCL | Cavity | 0.1 | 0.127 | NA | Negative | 1.0 | Species positive (single) | Possible IA (possible IA) |
| 60 | AML | No lesions | 0.1 | 0.114 | NA | NA | NA | NA | Unclassifiable |
| 61 | MCL | Halo sign, lobar infiltrate | 0.1 | 0.113 | Negative | Negative | 0.4 | Species negative | Possible IA (possible IA) |
| 62 | CML | Halo sign | 0.1 | 0.108 | NA | Negative | 0.1 | NA | Possible IA (possible IA) |
| 63 | Blastic lymphoma | Halo sign | 0.1 | 0.106 | Negative | Negative | 3.3 | Positive in duplicate | Probable IA (probable IA) |
| 64 | MM | Ground glass opacities | 0.1 | 0.106 | Negative | Negative | 0.1 | Species negative | Unclassifiable |
| 65 | ALL | No lesions | 0.1 | 0.106 | NA | NA | NA | NA | Unclassifiable |
| 66 | AML | Halo sign, wedge-shaped consolidation | 0.1 | 0.103 | Negative | Negative | 1.0 | Positive in duplicate | Probable IA (probable IA) |
| 67 | MDS | Nodules | 0.0 | 0.163 | Negative | Negative | 0.1 | Species negative | Possible IA (possible IA) |
| 68 | AML | Halo sign | 0.0 | 0.155 | Negative | Negative | 0.2 | Positive in duplicate | Probable IA (probable IA) |
| 69 | DLBCL | Ground glass opacities | 0.0 | 0.144 | NA | Negative | 0.1 | Species positive (single) | Unclassifiable |
| 70 | MDS | Cavity | 0.0 | 0.136 | NA | Negative | 0.1 | Positive in duplicate | Probable IA (probable IA) |
| 71 | AML | Halo sign | 0.0 | 0.134 | Positive | *A. fumigatus* | 0.1 | Species negative | Proven IA |
| 72 | AML | Halo sign | 0.0 | 0.128 | Negative | Negative | 0.4 | Species negative | Possible IA (possible IA) |
| 73 | MDS | Nodules | 0.0 | 0.127 | Negative | Negative | 0.1 | Species negative | Possible IA (possible IA) |
| 74 | MM | Halo sign | 0.0 | 0.118 | Negative | Negative | 0.2 | Species negative | Possible IA (possible IA) |
| 75 | AML | Halo sign | 0.0 | 0.111 | NA | Negative | 0.0 | Species negative | Possible IA (possible IA) |
| 76 | AML | Halo sign | 0.0 | 0.104 | Negative | Negative | 0.1 | Species positive (single) | Possible IA (possible IA) |
| 77 | PTLD after aHSCT for aplastic anemia | No lesions | 0.0 | 0.102 | NA | NA | NA | NA | Unclassifiable |

**Supplementary Table 1C. Details of 37 additional cases in which discrepant results were found between serum ELISA (ODI<0.5) and serum CLIA (CI ≥0.100).**

Abbreviations: aHSCT = allogeneic hematopoietic stem cell transplantation ALL = acute lymphoblastic leukemia; AML = acute myeloid leukemia; BAL = bronchoalveolar lavage; CI = concentration index; CML = chronic myeloid leukemia; DLBCL = diffuse large B cell lymphoma; EORTC/MSGERC = European Organization for Research and Treatment of Cancer and the Mycoses Study Group Education and Research Consortium; GM = galactomannan; HLH = hemophagocytic lymphohistiocytosis; IA = invasive aspergillosis; MCL = mantle cell lymphoma; MDS = myelodysplastic syndrome; MM = multiple myeloma; NA = not available; NHL = non-Hodgkin lymphoma; ODI = optical density index; PCR = polymerase chain reaction; PTLD = post-transplant lymphoproliferative disorder.

|  | Cohort 1  Spearman’s ρ [IQR], n | Cohort 2  Spearman’s ρ [IQR], n | All patients  Spearman’s ρ [IQR], n |
| --- | --- | --- | --- |
| BAL GM ELISA and serum GM ELISA | 0.518 [384-0.630], 147 | 0.431 [0.212-0.610], 70 | 0.505 [0.395-0.600], 217 |
| BAL GM ELISA and serum GM CLIA | 0.336 [0.179-0.476], 147 | 0.409 [0.186-0.592], 70 | 0.397 [0.275-0.506], 217 |
| BAL GM CLIA and serum GM ELISA | 0.595 [0.344-0.767], 41 | 0.493 [0.077-0.763], 22^#^ | 0.528 [0.316-0.690], 63 |
| BAL GM CLIA and serum GM CLIA | 0.412 [0.110-0.644], 41 | 0.420 [-0.016-0.721], 22^##^ | 0.424 [0.190-0.612], 63 |

**Supplementary Table 2. Quantitative correlations between BAL galactomannan ELISA, BAL galactomannan CLIA, serum galactomannan ELISA and serum galactomannan CLIA for all patients in whom bronchoalveolar lavage was performed. All correlations were significant with p<0.001, except for # (p=0.020) and ## (p=0.052).**

Abbreviations: BAL = bronchoalveolar lavage; CLIA = chemiluminescent immunoassay; ELISA = enzyme-linked immunosorbent assay; GM = galactomannan.

| **Sample number** | **Original result CLIA (CI)** | **Serie 1: result CLIA (CI)** | | | | | **Serie 2: result CLIA (CI)** | | | | | **Coefficient of variation (%)** |
| --- | --- | --- | --- | --- | --- | --- | --- | --- | --- | --- | --- | --- |
|  |  | **Run 1** | **Run 2** | **Run 3** | **Run 4** | **Run 5** | **Run 6** | **Run 7** | **Run 8** | **Run 9** | **Run 10** |  |
| 1 | 2.441 | NA | NA | NA | NA | NA | 2.522 | 2.251 | 2.198 | 1.861 | 1.844 | 13.0% |
| 2 | 0.967 | NA | NA | NA | NA | NA | 1.516 | 1.007 | 1.545 | 1.309 | 1.451 | 19.7% |
| 3 | 0.180 | 0.097 | 0.081 | 0.112 | 0.090 | 0.086 | 0.034 | 0.041 | 0.067 | 0.020 | 0.047 | 57.4% |
| 4 | 0.165 | 0.316 | 0.214 | 0.218 | 0.226 | 0.214 | 0.149 | 0.132 | 0.228 | 0.172 | 0.279 | 26.0% |
| 5 | 0.157 | NA | NA | NA | NA | NA | 0.083 | 0.146 | 0.101 | 0.078 | 0.123 | 28.6% |
| 6 | 0.111 | NA | NA | NA | NA | NA | 0.013 | 0.015 | 0.023 | 0.010 | 0.021 | 121.0% |
| 7 | 0.103 | 0.114 | 0.062 | 0.082 | 0.058 | 0.077 | 0.021 | 0.040 | 0.035 | 0.040 | 0.035 | 49.8% |
| 8 | 0.102 | 0.253 | 0.202 | 0.204 | 0.173 | 0.202 | 0.093 | 0.168 | 0.147 | 0.098 | 0.189 | 31.0% |
| 9 | 0.098 | 0.204 | 0.125 | 0.169 | 0.124 | 0.200 | 0.097 | 0.186 | 0.136 | 0.097 | 0.130 | 28.7% |
| 10 | 0.023 | NA | NA | NA | NA | NA | 0.019 | 0.024 | 0.035 | 0.016 | 0.032 | 29.6% |
| 11 | 0.023 | NA | NA | NA | NA | NA | 0.018 | 0.027 | 0.031 | 0.013 | 0.031 | 30.5% |

**Supplementary Table 3.** Results of two series of repeated testing of selected patient samples from cohort 1. The coefficient of variation was computed as the standard deviation divided by the mean of the test results for every individual sample.

Abbreviations: CI = concentration index; CLIA = chemiluminescence immunoassay; NA = not assessed.

**
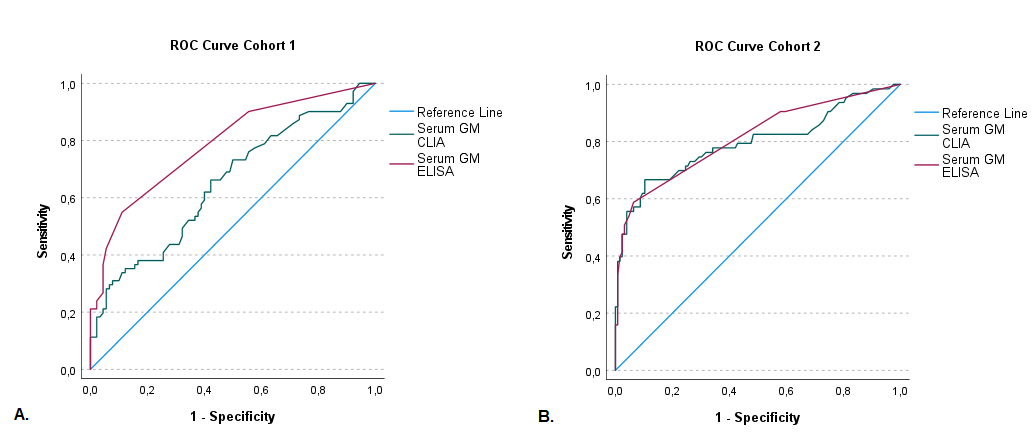
**

**Supplementary Figure 1. Panel A**: ROC curve for serum ELISA and serum CLIA of 161 serum samples from cohort 1 compared to the EORTC/MSGERC 2020 criteria. The area under the curve was 0.650 for CLIA and 0.787 for ELISA, p=0.005. **Panel B:** ROC curve for serum ELISA and serum CLIA of 189 serum samples from cohort 2 compared to the EORTC/MSGERC 2020 criteria. The area under the curve was 0.795 for CLIA and 0.813 for ELISA, p=0.649.
